# The relation between cognitive-perceptual schizotypal traits and the Ebbinghaus size-illusion is mediated by judgment time

**DOI:** 10.3389/fpsyg.2013.00343

**Published:** 2013-06-12

**Authors:** Paola Bressan, Peter Kramer

**Affiliations:** Department of General Psychology, University of PaduaPadua, Italy

**Keywords:** schizotypy, schizophrenia, magical ideation, Ebbinghaus illusion, contextual integration

## Abstract

In the Ebbinghaus illusion, a circle surrounded by smaller circles is perceived as larger than an identical one surrounded by larger circles. The illusion is reportedly weaker in individuals with (disorganized) schizophrenia or schizotypy than in controls, a finding that has been interpreted as evidence that both schizophrenia and schizotypy involve reduced contextual integration. In support of this view, we show that the Ebbinghaus illusion also decreases, in the general population, with cognitive-perceptual schizotypal traits (measured with both the cognitive-perceptual subscale of the *Schizotypal Personality Questionnaire-Brief* and the *Magical Ideation* scale). Our results were strong and separately replicable in different within-subjects and between-subjects conditions. However, a mediation analysis revealed that the reduction of the Ebbinghaus illusion was (statistically, hence without implying a causal relationship) entirely due to increased judgment time, i.e., the time subjects took to complete size comparisons. Judgment time increased with the strength of cognitive-perceptual schizotypal traits, but subjects with longer judgment times had smaller illusions regardless of these traits. We argue that there are at least two possible accounts of our results. Reduced contextual integration might be due to a reduced *ability* to integrate context, as previously suggested; alternatively, it could be due to a reduced *tendency* to integrate context—that is, to a detail-oriented processing style. We offer predictions for future research, testable with a deadline experiment that pits these two accounts against one another. Regardless of which account proves to be best, our results show that contextual integration decreases with cognitive-perceptual schizotypal traits, and that this relationship is mediated by judgment time. Future studies should thus consider either manipulating or measuring this time.

## Introduction

Schizophrenia is a heterogeneous mental disorder with various degrees of overlap with other diseases, such as bipolar disorder (Peralta and Cuesta, 2001; Dutta et al., 2007). It comprises several genetic, neurophysiological, and behavioral aspects (Lisman et al., 2008; Howes and Kapur, 2009; Javitt, 2009) whose co-occurrence, and relative dominance, vary between patients and over the course of the illness. Together, the behavioral aspects form a syndrome characterized by delusions (including paranoia), hallucinations, catatonia, psychomotor problems, and social dysfunction (Peralta and Cuesta, 2001). Schizotypy is a cognitive or biological vulnerability to schizophrenia that may or may not express itself clinically, and that affects people in the general population to various extents. To some, individuals with schizophrenia or schizotypy are categorically different from healthy ones (e.g., Meehl, 1990). To others, the three groups lie on one continuum (e.g., Claridge, 1994; McCreery and Claridge, 2002). In the current study, we will not commit to either view, but analyze quantitative differences between individuals as well as categorical ones.

There is ample evidence that schizophrenia-spectrum disorders are associated with a reduced consideration of context in both cognition and perception. Effects of reduced context processing have been found on sustained and selective attention, lexical disambiguation, latent inhibition, and size perception (for reviews, see Green et al., 2005; Silverstein and Keane, 2011), orientation perception (Yoon et al., 2009, 2010; Yang et al., 2013; but cf. Tibber et al., 2013), and early visual organization (Green et al., 2005)—more specifically on contour integration, perception of fragmented drawings, pattern recognition, grouping of dot patterns by proximity and color similarity, and coherent integration of different moving elements, such as in biological motion perception (Green et al., 2005; Kim et al., 2011; Silverstein and Keane, 2011).

With regard to contour integration, for example, some studies have investigated the detection of a low-contrast oriented grating (Gabor patch) flanked by two high-contrast ones that are either collinear or orthogonal to it (Must et al., 2004; Kéri et al., 2009, 2005; for related studies, see Keane et al., 2012; Halász et al., 2013). In non-patient controls, the collinear Gabor patches facilitate detection relative to the orthogonal ones. In patients with schizophrenia, instead, this effect was not found. The lack of facilitation in schizophrenia patients depends neither on medication, nor on insufficient attention to the task; these patients perform normally on an attentional control task with the same two flanking Gabors. In another study, a large set of small Gabor patches was presented in which some were oriented along a closed path and others were oriented randomly. Individuals with disorganized schizotypy (Uhlhaas et al., 2004) or disorganized schizophrenia (Uhlhaas et al., 2006a) detected the closed path less well than controls without schizotypy or schizophrenia. In an event-related-potentials study that used similar stimuli, both early and late processing components were implicated (Butler et al., 2013).

Related research investigated perceptual grouping by proximity and similarity. Schizophrenia patients were found to require a longer stimulus-duration than controls to perceive the grouping of little squares (Kurylo et al., 2007). A mask limited early perceptual processing, but not late decision making and response preparation. The observed effects thus appear to be bottom–up rather than top–down. In fact, at least the poorer grouping by proximity may be due to reduced sensitivity in low-spatial-frequency channels (the channels that pick up a blurred image of the stimulus) relative to high-spatial-frequency ones (O'Donnell et al., 2002).

Other studies have found superior performance among schizophrenia patients on tasks designed so that normal visual context processing would interfere with accuracy. In one such study, for example, the apparent contrast of a textured target surrounded by a high-contrast textured ring diminished, but only in patients without schizophrenia and in non-patient controls; patients with schizophrenia, instead, were not susceptible to this contrast-contrast effect (Dakin et al., 2005; see also Tibber et al., 2013; Yang et al., 2013; but cf. Barch et al., 2012). In two other studies, reports of the numerosity of a set of bars deteriorated with the orientation heterogeneity of the bars, but again only in patients without schizophrenia and in non-patient controls, and not in patients with schizophrenia (Schwartz Place and Gilmore, 1980; Wells and Leventhal, 1984). These findings of superior, rather than inferior, performance in schizophrenia patients more definitively exclude potential confounds related to motivation or a general difficulty in performing the task.

The reduced processing of context in schizophrenia has been argued to have a large impact and to lead to a cascade of other abnormalities. Indeed, impairments have been found in face perception, which arguably depends on visual organization (Green et al., 2005; Silverstein and Keane, 2011), and in theory-of-mind skills, which arguably depend both on the ability to interpret facial expressions and on memory for the particular circumstances (context) in which people operate (Green et al., 2005).

Still, there have also been failures to find reduced contextual effects in schizophrenia. For example, though some have found weaker contextual-integration effects on perceived motion (Tadin et al., 2006; Yang et al., 2013), others have found an abnormally strong one (Chen et al., 2008; see also Chen, 2011). The perception of shades of gray (lightness) is another case in point. It heavily depends on context (for reviews, see Gilchrist et al., 1999; Bressan, 2006; Kingdom, 2011) and typically a target region appears lighter if surrounded by a dim region than if surrounded by a bright one (although exceptions exist: e.g., Kramer and Bressan, 2010). This lightness-contrast effect, however, has not been found to differ between patients with schizophrenia and healthy individuals (Tibber et al., 2013; Yang et al., 2013), although—as mentioned earlier—the contrast-contrast effect does differ between the two groups.

**Figure 1:**
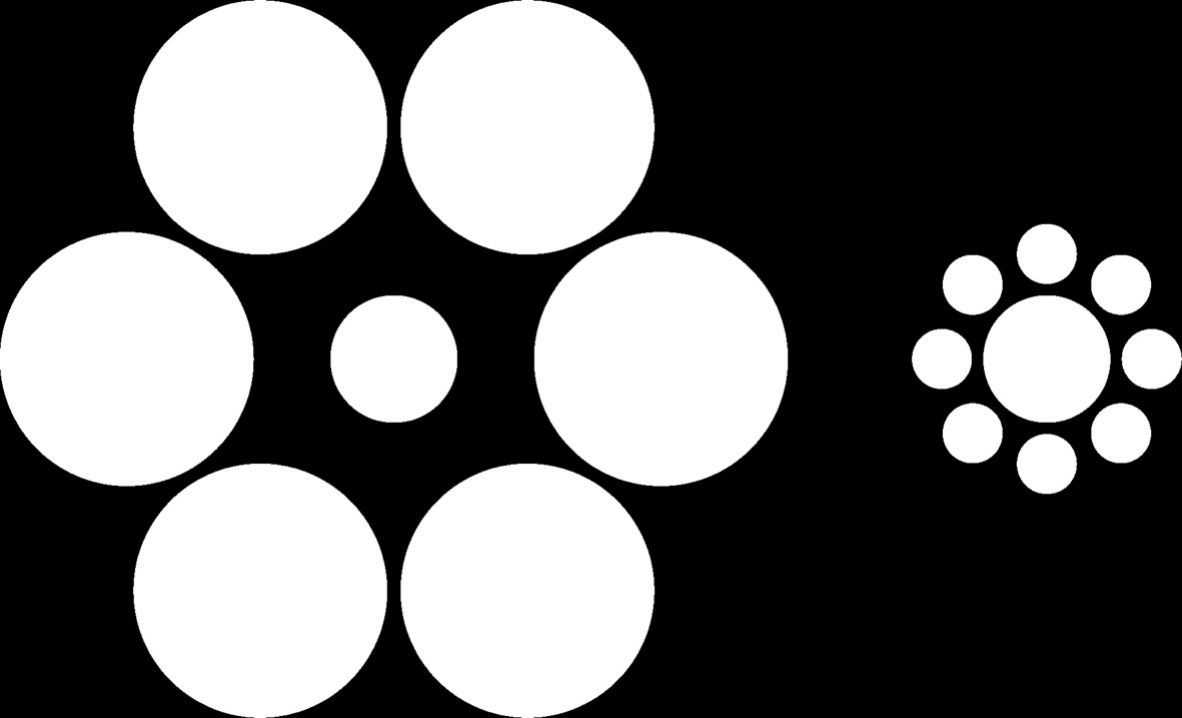
**Ebbinghaus illusion**. The central disk on the left appears smaller than the physically identical one on the right. (Similar effects are obtained with circles instead of disks.)

In the Ebbinghaus illusion, a circle surrounded by smaller circles is perceived as larger than an identical one surrounded by larger circles (Figure 1). The illusion is reportedly weaker in individuals with disorganized schizophrenia (Uhlhaas et al., 2006a) or disorganized schizotypy (Uhlhaas et al., 2004) than in controls without schizophrenia or schizotypy, a finding that, again, has been interpreted as evidence that both schizophrenia and schizotypy involve reduced contextual integration (see also Green et al., 2005; Silverstein and Keane, 2011). The reduction in the illusion has not been found in individuals with schizotypy (Uhlhaas et al., 2004) or schizophrenia (Uhlhaas et al., 2006a; Yang et al., 2013) lacking disorganization.

Because a smaller Ebbinghaus illusion amounts to a higher accuracy in size estimation, confounds related to insufficient motivation or reduced ability to perform the task are unlikely. In principle, however, it is possible that the strength of the Ebbinghaus illusion could vary with the time subjects take to compare the central circles (judgment time). Subjects relying on a quick glance at the stimulus, for example, may perceive it differently than those who inspect it more in detail (van Zoest and Hunt, 2011). Some studies showed that size judgments of attended compared to unattended objects were more accurate (Epstein and Broota, 1986; Prinzmetal and Wilson, 1997), whereas another study found that attended lines appeared slightly longer than unattended ones (Masin, 2008). Indeed, attention has also been found to modulate the Ebbinghaus illusion itself (Shulman, 1992). Due to the potentially confounding effect of either method- or self-imposed limitations on judgment time—stemming, for example, from fixed presentation times or large numbers of trials—the effects on the illusion of schizophrenic or schizotypal traits other than disorganization may have escaped detection and prove moderated or mediated by this variable.

In the current study, we investigate this possibility. More specifically, taking judgment time into account, we test whether the Ebbinghaus illusion decreases with cognitive-perceptual schizotypal traits. To accomplish this, we use a relatively large sample of individually-tested ordinary subjects (mostly psychology students), who are expected to express varying degrees of schizotypal characteristics. First, we investigate whether the Ebbinghaus illusion diminishes with increasing scores on the cognitive-perceptual subscale of Raine and Benishay's (1995) *Schizotypal Personality Questionnaire-Brief* and, as a separate control, with increasing scores on Eckblad and Chapman's (1983) *Magical Ideation* scale, that measures similar aspects of cognitive-perceptual schizotypy with different items. And second, although—as in related previous studies—we do not urge subjects to be fast, for the first time we measure how long it takes them to complete their task.

## Materials and methods

### Participants

A total of 123 naïve (mostly psychology) students of the University of Padua volunteered to participate in the study (83 women and 40 men; median age 23, age range 20–30 years). They were requited and tested individually. The experimental procedures were approved by the Institutional Review Board at the University of Padua, and were in accordance with the Declaration of Helsinki (Sixth Revision, 2008). All participants gave their informed written consent to participate in the study.

### Apparatus, stimuli, and procedure

Stimuli were presented with the help of a personal computer and a custom E-Prime (Psychology Software Tools, Inc.). program on a calibrated Quato Perfect Color 22-inch CRT monitor, shielded by a custom black hood and placed in a totally dark laboratory. Response times were recorded with millisecond precision. Viewing distance was about 57 cm (measures reported here in centimeters were therefore roughly equivalent to measures in degrees of visual angle). Two white disks were presented in the centers of two abutting black backgrounds (15.5 × 15.5 cm). On half of the trials, eight small disks (1.1 cm in diameter) surrounded the left disk and six large ones (4.8 cm in diameter) the right disk (Figure 1). On the other half of the trials, the left and right stimuli were reversed. With a 1 × 1 cm cursor, placed within a 26.5 × 1 cm slide centered 3 cm below each stimulus pair, subjects modified the size of the right disk until it matched that of the left disk (2.3 cm in diameter). Subjects finalized their response with a space-bar press. The adjustable disk started very small, so that it had to be increased, on half the trials, and very large, so that it had to be decreased, on the other half. The combination of adjustable disk's context (small, large) and initial size (very small, very large) gave a total of four conditions, presented once to each subject and in the same order to all. (For a purpose related to a separate study, concurrently run with the present one, the stimuli were preceded by others in which the same method of adjustment was used, and the rest of the screen was either blue or red).

The left and right halves of the Ebbinghaus stimuli each contribute to the Ebbinghaus illusion (Franz et al., 2000). However, their relative contributions are not comparable. In our stimuli, for example, the distance between the inner edges of the contextual and target disks is smaller for the small contextual disks than for the large ones. In a comparison between the contributions of the left and right sides, this difference in edge-to-edge distance would be a confound. It is impossible to eliminate this confound without introducing another (Rose and Bressan, 2002). Thus, here we only consider the size of the entire Ebbinghaus illusion and not of its parts.

### Materials

After the experiment, participants filled out our Italian translations of (1) Eckblad and Chapman's (1983) *Magical Ideation scale* (*MI*) and (2) the cognitive-perceptual subscale of Raine and Benishay's (1995) brief version of the *Schizotypal Personality Questionnaire* (*SPQB*) (Raine, 1991). The first author translated the scales from English into Italian, the second author—blind to the originals—translated them back into English. An independent reviewer checked the back-translations' accuracy; no noteworthy differences were found. The authors and the reviewer are all fluent in both English and Italian and have extensive translation experience.

The MI scale measures cognitive-perceptual aspects of schizotypy and consists of 30 items that require a true or false response. Example items are: “I have occasionally had the silly feeling that a TV or radio broadcaster knew I was listening to him”; “Numbers like 13 and 7 have no special powers” (scored negatively); “I have felt that I might cause something to happen just by thinking too much about it.” The SPQB's cognitive-perceptual (SPQB-CP) subscale measures the same aspects and consists of 8 items that require a yes or no response. Example items are: “Do you often pick up hidden threats or put-downs from what people say or do?”; “Have you had experiences with astrology, seeing the future, UFOs, ESP, or a sixth sense?”; “Have you ever had the sense that some person or force is around you, even though you cannot see anyone?”. The SPQB-CP subscale and the MI scale measure similar aspects of schizotypy, allowing us to cross-validate them within our sample.

### Statistics

One of the analyses we performed was a mediation analysis (Baron and Kenny, 1986), an analysis that establishes whether or not the correlation between one variable and another is statistically due to their correlation with a third variable. Note that the third variable need not necessarily be the cause of the correlation between the first two variables, because one or more variables that are not considered could play that role instead.

In order to confirm that a third variable (here judgment time) mediates the relationship between an independent variable (here either SPQB-CP or MI scores) and a dependent one (here Ebbinghaus-illusion magnitude), it has to be established that (1) the independent variable correlates significantly with the dependent variable, (2) the independent variable correlates significantly with the third variable, and (3) the third variable correlates significantly with the dependent variable, even after controlling for the independent variable. That is, in a multiple regression, the contribution of the independent variable should be greatly reduced in the presence of the third variable and ideally become non-significant. (If the contribution of the independent variable remains significant and considerable, but does depend on the third variable, then the latter is a moderator rather than a mediator.)

## Results

One data point corresponded to an Ebbinghaus illusion that was more than three standard deviations below the mean, and one data point to a response time that was more than three standard deviations above it. Although their inclusion led to virtually identical results, these two data points were excluded from analysis.

In the Ebbinghaus illusion, the circle surrounded by smaller circles is perceived as larger than the identical one surrounded by larger circles (Figure 1). Thus, if subjects are to adjust the former's size so that it matches the latter's, they should have a tendency to adjust downward. In the converse case, they should have a tendency to adjust upward. Indeed, when the fixed disk in our experiment was surrounded by larger ones and the adjustable disk by smaller ones, subjects reduced the adjustable disk's size by 16% (SD 8%). Conversely, when the fixed disk was surrounded by smaller ones and the adjustable disk by larger ones, subjects enlarged the adjustable disk's size by 24% (SD 11%). The mean Ebbinghaus-illusion magnitude was thus 20% (SD 8.4%). The average time taken to complete the adjustment (judgment time) was 7.7 s (SD 6.1 s). To eliminate skew in judgment times, we log-transformed them, but the transformed and untransformed data produced almost identical results.

**Figure 2:**
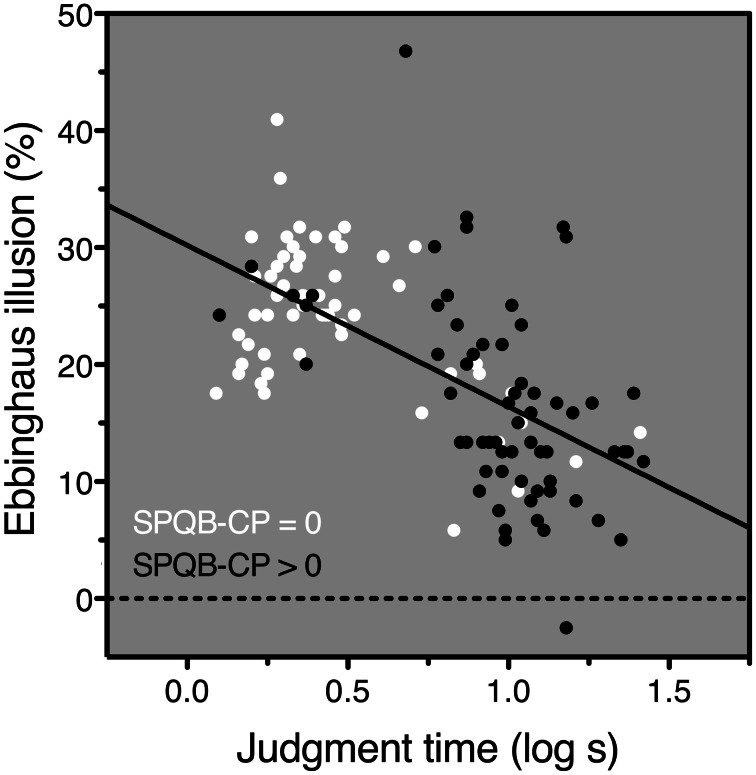
**Results**. Ebbinghaus-illusion magnitude plotted against log-transformed judgment time. Subjects with an SPQB-CP score of 0 are represented in white; those with an SPQB-CP score larger than 0 are represented in black. The regression line is a fit to all data points regardless of SPQB-CP score (i.e., regardless of symbol color). Note that individuals with a positive SPQB-CP score tend to be less susceptible to the Ebbinghaus illusion, but to have a longer judgment time, and that regardless of SPQB-CP score, individuals with a longer judgment time tend to be less susceptible to the Ebbinghaus illusion.

The SPQB-CP scores ranged from 0 to 8 and had a mean of 1.6 (SD 2.0); nearly half the subjects (47.2%) scored 0. Because the distribution was so heavily skewed, we performed all analyses both on the raw SPQB-CP scores and on a dichotomized variable on which scores were categorized as either “zero SPQB-CP” or “positive SPQB-CP” (above 0). Still, results in the two cases were quite similar, and hence, we only report the former. For better visualization, in Figure 2 we show the dichotomized data.

We found that the Ebbinghaus-illusion magnitude decreased with SPQB-CP scores, but the effect was entirely mediated (Baron and Kenny, 1986) by judgment time (Figure 2): (1) Ebbinghaus-illusion magnitude decreased with SPQB-CP scores: *r* = −0.41, *p* < 0.0001, *N* = 122; (2) SPQB-CP scores increased with judgment time: *r* = 0.64, *p* < 0.0001, *N* = 122; (3) in a multiple regression that explained 38% of the Ebbinghaus-illusion variance: *R* = 0.62, *F*_(2, 118)_ = 36.5, *p* < 0.0001, only the judgment-time coefficient was significant: β = −0.60, *t* = −6.28, *p* < 0.0001, whereas the SPQB-CP coefficient did not even reach marginal significance: β = −0.03, *t* = −0.36, *p* = 0.716. Age correlated with SPQB-CP and judgment time, but not with Ebbinghaus-illusion magnitude: respectively, *r* = −0.20, *p* = 0.030, *N* = 123, *r* = −0.20, *p* = 0.027, *N* = 122, *r* = 0.02, *N* = 122; adding age to the multiple regression model left the results virtually unchanged.

Taking SPQB-CP out of the multiple regression model, the Ebbinghaus illusion still decreased with judgment time: *r* = −0.62, *p* < 0.0001, *N* = 121. Moreover, it did so not only in subjects whose SPQB-CP score was positive: *r* = −0.50, *p* < 0.0001, *N* = 64, but also in those whose SPQB-CP score was 0: *r* = −0.52, *p* < 0.0001, *N* = 57.

The MI scores ranged from 0 to 23 and had a mean of 6.0 (SD 4.0). MI and SPQB-CP scores were highly correlated, *r* = 0.74, *N* = 123, *p* < 0.0001, and the MI results followed the same pattern as the SPQB-CP results. We found that (1) Ebbinghaus-illusion magnitude decreased with MI: *r* = −0.19, *p* = 0.037, *N* = 122, (2) MI increased with judgment time: *r* = 0.38, *p* < 0.0001, *N* = 122, and (3) in a multiple regression that explained 38% of the Ebbinghaus-illusion variance: *R* = 0.62, *F*_(2, 118)_ = 36.9, *p* < 0.0001, only the judgment-time coefficient was significant: β = −0.64, *t* = −7.78, *p* < 0.0001, whereas the MI coefficient did not even reach marginal significance: β = 0.07, |*t*| < 1. Age did not correlate with MI.

Demonstrating within-subjects replicability, the Ebbinghaus illusion decreased with judgment time, and separately also with SPQB-CP scores, both when the adjustable disk was surrounded by smaller disks and when it was surrounded by larger ones (all *p*s < 0.0001). Demonstrating between-subjects replicability, the Ebbinghaus illusion decreased with judgment time in both men and women (both *p*s < 0.0001). The illusion also separately decreased with SPQB-CP scores, significantly in the relatively large sample of women (*p* < 0.0001; *N* = 82) and marginally so in the smaller sample of men (*p* = 0.09; *N* = 40).

## Discussion

In a relatively large sample taken from the general population, we found that the Ebbinghaus illusion decreases with cognitive-perceptual schizotypal traits. Unlike in earlier related studies, however, we measured the time subjects took for their size judgment and found that the reduction in the illusion was (statistically, hence without implying a causal relationship) entirely mediated by this judgment time. Before examining possible explanations of our results, let us first discuss how our study compares to previous ones.

### Comparison with previous studies

First, the Ebbinghaus illusion has previously (Uhlhaas et al., 2004) been reported to decrease with disorganization traits in schizotypy, but not with other schizotypal traits. In our study, we presented a fairly strong version of the Ebbinghaus illusion, similar to the classic one—with small inducing disks around one target and large inducing disks around the other (Figure 1). Instead, Uhlhaas et al. (2004)—and also Yang et al. (2013), who failed to find an effect of schizophrenia on the illusion—used a version of the Ebbinghaus illusion in which only one half of the stimulus was presented along with a neutral condition, a version known to produce an illusion less than half as large as the classic one (Franz et al., 2000). This choice of stimuli may thus have reduced power. Indeed, in Uhlhaas et al. (2004) the results of individuals with disorganized schizotypy were significantly different from those of individuals with non-disorganized schizotypy, and from controls without schizotypy, only when the illusion-inducing context consisted of large disks and not when it consisted of small ones.

Second, Uhlhaas et al. (2006a) reported that the Ebbinghaus illusion decreased in patients with disorganized schizophrenic traits but not in patients with other schizophrenic traits; however, no significant difference was found *between* these two conditions. Hence, the results do not allow the conclusion that the Ebbinghaus illusion is affected differently by disorganized schizophrenic traits and by cognitive-perceptual ones. Horton and Silverstein (2011) did find a significant difference between these conditions, but only for deaf patients, and they were not compared to individuals without schizophrenia. For normally hearing patients—who were not compared to individuals without schizophrenia either—the difference was significant only when the illusion-inducing context consisted of small disks and not when it consisted of large ones; a result opposite to that found for schizotypy by Uhlhaas et al. (2004).

Third, in our main analyses, unlike in the earlier study on schizotypy and the Ebbinghaus illusion by Uhlhaas et al. (2004), we did not cluster subjects into an experimental and a control group, but maintained the raw questionnaire scores. Thus, we preserved information about, for example, differences between subjects scoring low and subjects scoring 0 on the SPQB-CP. With 47.2% of our sample scoring indeed 0, this preservation of information may have been important.

Fourth, we used a different method from previous related studies: the method of adjustment rather than a forced choice. One possible consequence of our method is that our subjects might have taken longer to perform their task than the subjects of earlier studies. Because previous studies of the Ebbinghaus illusion did not measure response time, it is difficult to verify this. Still, Uhlhaas et al. (2006a)—who did not investigate response time either—reported that their Ebbinghaus stimuli were presented for 4 s. Assuming that subjects responded before or immediately after stimulus offset, they were likely to have responded faster than our subjects (whose average response time was 7.7 s). It therefore remains unclear whether effects of cognitive-perceptual schizotypal traits could have emerged if presentation, and response, times had been unlimited. Notably, Uhlhaas et al. (2006b), who did investigate response time and also perceptual organization, but not the Ebbinghaus illusion, found that patients with schizophrenia had longer response times than healthy controls.

### Possible explanations of our results

The differences between the current and previous studies were thus large enough to explain how we could have obtained different results. The question now is their explanation. Various studies use tasks that require contextual integration. A reduced ability, or reduced tendency, to perform this integration is then likely to increase response time, decrease accuracy, or both (e.g., Uhlhaas et al., 2006b). In studies like ours that use a size-judgment task, contextual integration is not required, but our data suggest that it is nevertheless, to various extents, performed by almost all subjects (Figure 2). It is thus possible that subjects with a reduced ability to integrate context lose time doing it. This conjecture is consistent with our finding that the Ebbinghaus illusion, which depends on contextual integration, decreases with judgment time. We also found that the Ebbinghaus illusion decreases with cognitive-perceptual schizotypal traits and this effect could be due to a negative relationship between these traits and the ability to integrate context. However, because we found that the Ebbinghaus illusion decreases with judgment time *regardless* of these traits, our results suggest that the link between reduced contextual integration and these traits cannot be an exclusive one.

A reduced Ebbinghaus illusion amounts to increased size-judgment accuracy. As discussed in our introduction, this fact has been used to argue that a reduced Ebbinghaus illusion is unlikely to be due to inattention or weaker motivation. Yet, it is still possible that some subjects base their judgment on a quick global impression of the stimulus, whereas others go through the trouble of inspecting it in detail. The processing of a stimulus's details generally takes longer than the processing of its global properties (for a review, see Kimchi, 1992). In addition, subjects performing their size judgments for a longer time focus, necessarily, more on the task-relevant targets and less on the task-irrelevant context. As unattended context affects the Ebbinghaus illusion less than attended context (Shulman, 1992), one would thus expect the illusion to decrease with judgment time. Indeed, this is what we found. The remaining question is whether the tendency to inspect the stimulus in detail, or to inspect *any* stimulus in detail (i.e., the tendency to rely on a detail-oriented processing style), may somehow be related to schizotypal traits (see also Phillips et al., 2004; Doherty et al., 2010).

One possible relation comes to mind when one considers that schizotypy and schizophrenia—and especially their positive symptoms that we have measured here—are often comorbid with anxiety and negative mood (e.g., Lewandowski et al., 2006). It has been argued that these moods induce a detail-oriented processing style, possibly via a reduction in the scope of attention (the so-called Easterbrook hypothesis: Easterbrook, 1959; for a review, see Friedman and Förster, 2010). Although a few studies have failed to find evidence for effects of mood on perceptual organization, many have corroborated it.

Among the former are some studies by Silverstein and colleagues. Silverstein et al. (1992), for example, found no effect on perceptual organization of either depression or anhedonia. Likewise, Silverstein et al. (1996) found no effect on perceptual organization of belonging to a control group of individuals with either depression accompanied by psychotic features, bipolar disorder accompanied by psychotic features, schizoaffective disorder, or delusional disorder. Uhlhaas et al. (2006a), finally, found no effect on the Ebbinghaus illusion of belonging to a control group that included depressed individuals; in this group, however, the depressed individuals were a minority.

A large number of studies by different research groups have found evidence that both anxiety and negative mood do promote detail-oriented processing. The tendency, for example, to classify large shapes consisting of small shapes (Navon, 1977; Kimchi, 1992) on the basis of the small shapes, rather than the large ones, was found to increase with both trait anxiety (Tyler and Tucker, 1982) and negative mood (Basso et al., 1996; Gasper and Clore, 2002; Gasper, 2004; Fredrickson and Branigan, 2005). Likewise, responses to central targets were hampered less by flanking irrelevant context (Eriksen and Eriksen, 1974) when mood was negative than when it was neutral or positive (Fenske and Eastwood, 2003; Rowe et al., 2007; Schmitz et al., 2009; Moriya and Nittono, 2011). Consistent evidence has also been found with various other techniques, ranging from the detection of peripheral targets (Weltman et al., 1971) to the holistic recognition of faces (Curby et al., 2012). [For further evidence, discussion, and the latest theoretical refinements, see Friedman and Förster (2010), and Huntsinger (2012).]

### Predictions for future research

In our view, there are thus at least two possible accounts of our results: one in which reduced contextual integration is due to a reduced *ability* to integrate context and one in which it is due to a reduced *tendency* to integrate context—that is, to a detail-oriented processing style. Given that a processing tendency should be more flexible than a processing disability, with a deadline experiment (an experiment in which subjects are forced to respond before a variable deadline; e.g., Kramer et al., 2013) these two accounts can be pitted against each other. At a long deadline, some may have a large Ebbinghaus illusion and some may have a small one. As the deadline decreases, however, the reduced-tendency account predicts that the two groups should become more similar, whereas the reduced-ability account predicts they should not. That is, according to the reduced-tendency account, time pressure should redirect subjects away from a time-consuming detailed analysis of the stimulus and toward a quick judgment based on a short glance at it. Stated differently, time pressure should redirect subjects' orientation from the trees (i.e., details) to the forest (i.e., the big picture). (For similar ideas see, e.g., Tyler and Tucker, 1982.) According to the reduced-ability account, this redirection should not occur, because those who integrate little of the context at long deadlines should not be able to integrate more of it at short ones.

## Conclusion

Regardless of which account turns out to be best, our present results challenge previous claims that contextual integration does not decrease with cognitive-perceptual schizotypal traits, but also show that this relationship (again statistically, and hence without implying a causal relationship) is entirely mediated by judgment time. Future studies should thus consider either manipulating or measuring this time.

### Conflict of interest statement

The authors declare that the research was conducted in the absence of any commercial or financial relationships that could be construed as a potential conflict of interest.
